# Bioprospecting Pantanal Aquatic Macrophytes as Sources of Plant-Derived Biostimulants for Soybean Seed Treatment

**DOI:** 10.3390/plants15213429

**Published:** 2026-11-04

**Authors:** Jailson Vieira Aguilar, Suellen Rodrigues Ramalho, Alana Martini Ferreira, Kamila De Sousa Gomes, Jessica Carolina Medina-Gallardo, Karenn Gianotti Franco Bastos, Gisele Marta Martins, Bruno Cesar Da Silva Coelho, Diego Campos Vasconcelos, Leandro da Conceição, Layssa Aline Okamura, Desiree Soares da Silva

**Affiliations:** 1 SENAI Innovation Institute for Biomass, Três Lagoas 79640-250, Mato Grosso do Sul, Brazil; suellen.ramalho@ms.senai.br (S.R.R.); alanaferreira.isi@ms.senai.br (A.M.F.); kamila.gomes@ms.senai.br (K.D.S.G.); jessica.gallardo@ms.senai.br (J.C.M.-G.); karenn.bastos@ms.senai.br (K.G.F.B.); gisele.martins@ms.senai.br (G.M.M.); bruno.coelho@ms.senai.br (B.C.D.S.C.); leandro.conceicao@ms.senai.br (L.d.C.); l.okamura@ms.senai.br (L.A.O.); desiree.silva@ms.senai.br (D.S.d.S.); 2 SENAI Regional Department of Mato Grosso do Sul, Campo Grande 79005-901, Mato Grosso do Sul, Brazil; diego.vasconcelos@ms.senai.br

**Keywords:** aquatic macrophytes, pantanal, plant extracts, plant biostimulant, *Glycine max*, seed germination, seedling growth

## Abstract

The Pantanal biome harbors a high diversity of aquatic macrophytes that remain largely unexplored as potential sources of plant-derived agricultural biostimulants. This study evaluated six aquatic macrophytes from the Pantanal: *Eleocharis cellulosa*, *Pistia stratiotes*, *Thalia geniculata*, *Pontederia* sp., *Pontederia rotundifolia*, and *Pontederia azurea* as potential sources of plant-derived biostimulants. Aqueous and methanolic extracts were prepared by dynamic maceration and tested on soybean seed treatment. FTIR spectroscopy was used to characterize functional groups. Final germination percentage ranged from 86% to 97% across treatments, and no extract significantly reduced this endpoint compared with the respective controls, indicating the absence of detectable phytotoxic effects under the tested conditions. *P. rotundifolia*, *T. geniculata*, and *P. azurea* promoted increases in biomass and length (up to 0.44 g of epicotyl dry mass and 14.62 cm of root length), emerging as promising biostimulant source. *E. cellulosa* and *Pontederia* sp. showed neutral or negative effects on most growth variables. *P. stratiotes* remained neutral. FTIR analysis revealed distinct spectral profiles between extraction methods. Aqueous extracts showed prominent absorption bands associated with carbohydrate and polysaccharide-related functional groups, whereas methanolic extracts exhibited additional bands consistent with phenolic, aromatic, ester, and lipid-associated functional groups. The aqueous route stood out for producing bioactive extracts without organic solvents, a key advantage for sustainable scaling.

## 1. Introduction

The contemporary global agricultural landscape faces a fundamental paradox, ensuring food security for a growing population while mitigating the environmental impacts of conventional production models. Climate change intensifies extreme events such as prolonged droughts, heatwaves, and soil salinization factors that threaten to drastically reduce arable land by 2050 [1]. Concurrently, the intensive use of chemical fertilizers and synthetic pesticides has contributed to soil degradation, water contamination, and biodiversity loss [2]. Sustainable agricultural intensification therefore increasingly depends on strategies capable of improving crop productivity while reducing environmental impacts [3].

Agricultural bioinputs have emerged as key players in this transformation, offering nature-based alternatives for plant nutrition, protection, and development. Bioinputs encompass biologically derived products, including microorganisms, plant extracts, and other natural substances, which may improve nutrient-use efficiency, plant growth, stress tolerance, or crop protection [4]. The global market is expanding significantly, driven by demand for sustainable practices and reduced reliance on chemical inputs [5,6]. Brazil has emerged as one of the global leaders in both innovation and adoption of these technologies, reflecting strong sector dynamism and consistent growth [7]. The recent approval of Law 15.070/2024 [8] further consolidates a favorable environment for research and innovation.

Despite the sector’s rapid growth, important scientific challenges remain concerning efficacy validation, product standardization, regulatory registration, and elucidation of mechanisms of action. Among bioinput categories, biostimulants represent the fastest-growing segment, driven by the need to manage abiotic stresses such as drought, salinity, and extreme temperatures [9,10]. Plant-derived bioactive compounds, including hormones, amino acids, polysaccharides, and phenolics, can trigger physiological responses that enhance crop tolerance to adverse conditions [11]. However, research on plant-derived biostimulants has focused predominantly on terrestrial plant species and marine macroalgae, whereas freshwater aquatic macrophytes remain comparatively underexplored. In particular, the biostimulant potential of aquatic macrophytes from tropical wetlands such as the Pantanal has received limited systematic investigation [9,12,13]. This gap is both taxonomic and geographic, few studies have systematically evaluated continental aquatic plants for biostimulant activity, and the Pantanal region, one of the most biodiverse ecosystems on the planet, remains particularly insufficiently characterized in this context.

This biome, with its unique biodiversity and extreme hydrological regimes, harbors species adapted to seasonal flooding and environmental stress, representing a poorly investigated source of metabolites with potential agricultural applications [14]. Harsh environmental conditions are recognized to favor physiological adaptations that may promote the biosynthesis of secondary metabolites in plants [15], thereby increasing the phytochemical richness of native aquatic species. Phenolic compounds, flavonoids, and terpenoids well known for their antioxidant and plant growth-regulating properties are commonly associated with stress-adapted species and therefore represent plausible candidates to occur in Pantanal plants. These floodplain areas offer a strategic model system for applying an integrated bioprospecting approach, given their extreme hydrological regimes, rich aquatic flora, and the potential of their active constituents for agricultural bioinput development.

However, exploration of this still poorly studied biodiversity requires robust methodologies to ensure reproducibility and reliable characterization of candidate species. From field collection and taxonomic authentication to extract preparation and chemical characterization, each step must be carefully executed to ensure that functional potential is reliably assessed. Molecular identification provides taxonomic precision [16], solvent selection directly influences the recovery of active compounds [17], and FTIR spectroscopy provides rapid information on the major functional groups present in complex plant extracts [18]. When combined with standardized bioassays following the Brazilian Rules for Seed Analysis [19], these complementary approaches allow meaningful evaluation of biostimulant activity, increasing the likelihood of identifying effective candidates for agricultural application [20].

In response to this gap, the present study proposes an innovative approach to bioprospecting aquatic plants from the Pantanal for agricultural bioinput development. We hypothesized that aquatic macrophytes from the Pantanal contain extractable compounds capable of modulating soybean germination and early seedling growth and that these responses vary according to plant species and extraction solvent. Therefore, this study aimed to evaluate six Pantanal aquatic macrophytes through molecular identification, aqueous and methanolic extraction, FTIR-based characterization, and soybean seed bioassays to identify promising species and extraction routes for further biostimulant development.

## 2. Results

### 2.1. Yield of Processed Collected Plant Material

After collection and molecular identification of the biomass obtained from the Pantanal biome, the samples were dried, milled, and stored for subsequent analyses. Sample processing data are presented in Table 1.

Total dry biomass of each species ranged from 1.22 kg (*Pontederia* sp.) to 6.66 kg (*Thalia geniculata*). The value of 6.66 kg was notably higher, with an absolute increase of 3.40 kg and a percent difference of 104.2% compared to the second highest dry biomass collected (3.26 kg for *Eleocharis cellulosa*). This elevated value was unique, as the remaining samples were closer to the lower end of 1.22 kg (Table 1).

After knife milling, biomass underwent a slight decrease due to losses associated with sample handling and processing, which is expected and standard in this type of activity. The milling yield ranged from 88.96% for *E. cellulosa* to 95.15% for *Pistia stratiotes*, with intermediate values for *T. geniculata*, *Pontederia* sp., and *Pontederia azurea*. Mean milling yield across species was approximately 91.4%. The visual appearance of the milled biomass of each species is shown in Figure S1 in the Supplementary Material.

In addition to biomass yield, the residual moisture content of the oven-dried, milled plant material was evaluated for all species prior to extraction. The residual moisture ranged from 4.18% (*P. azurea*) to 8.41% (*P. stratiotes*) (Table 1).

### 2.2. Aqueous and Methanolic Extracts Yield and Retention

The mass balance, represented by extraction yield and retention values for both types of extracts, is presented in Table 2. The aqueous extract yield showed low variation, as indicated by the standard deviation (*n* = 3). The highest deviations were observed for *Pontederia rotundifolia* (±0.93) and *Pontederia azurea* (±0.88), while the lowest deviation was observed for *Eleocharis cellulosa* (±0.18). Although numerically the values fall within this range of variation, the data context justifies the deviation, as the highest standard deviation was also observed for the highest yield value (21.37%). The remaining yield values ranged from 8.82% (*E. cellulosa*) to 14.96% (*Pontederia* sp.) (Table 2).

Retention complements yield, so that a high yield value is accompanied by a low retention value, while the opposite can also be observed. In this context, the lowest retention was found for *P. rotundifolia*. Unlike yield, retention showed wider standard deviation values, indicating greater variation for some species. The triad *E. cellulosa*, *P. stratiotes*, and *T. geniculata* had similar deviations, ranging from 2.05 to 2.66%, whereas the other three species showed notably lower deviations, from 0.14 to 0.66%.

For all species, methanolic extract yield was lower than aqueous extract yield. Among the methanolic extracts, the highest yield was observed for *P. azurea* (10.91%), while *T. geniculata* and *P. rotundifolia* showed values close to the highest, 10.11% and 10.07%, respectively. The other three species had yields ranging from 4.16% to 5.11%.

Overall, deviations were low and uniform, ranging from 0.11% to 0.61%. The deviation observed for *E. cellulosa* extract (0.57%) was the most expressive considering its low yield value (4.84%), compared to the highest deviation observed for *T. geniculata* extract. Retention values and deviations of methanolic extracts were also uniform and consistent with the yield-retention relationship. The appearance of the extracts immediately after drying is shown in Figure S2 in the Supplementary Material.

### 2.3. FTIR Spectroscopic Characterization

Table 3 presents the main FTIR absorption bands for the aqueous and methanolic extracts of the six species. In the aqueous extracts, all samples exhibited a broad and intense band between 3254 and 3281 cm^−1^, corresponding to O-H stretching, and a strong band between 1007 and 1072 cm^−1^, attributed to C-O stretching.

These bands are consistent with O–H and C–O-containing functional groups commonly associated with carbohydrate-rich matrices, including polysaccharides. Additionally, medium-intensity bands from 1587 to 1637 cm^−1^, attributed to overlapping C=O and C=C stretching. These bands varied among species: in *Eleocharis cellulosa* and *Pistia stratiotes*, they appeared at 1621 and 1637 cm^−1^, respectively, suggesting the presence of carbonyl groups, possibly from amides or conjugated carboxylic acids; in the three *Pontederia* species and *Thalia geniculata*, these bands were observed at 1587–1608 cm^−1^, suggesting a greater contribution from aromatic rings and phenolic compounds in the aqueous fraction.

In the methanolic extracts, the O-H and C-O bands were also present in all species (3234–3382 cm^−1^ and 1028–1076 cm^−1^), indicating that residual carbohydrates were co-extracted by methanol. However, methanol extraction resulted in more pronounced differences in chemical composition among species.

The methanolic extracts of *E. cellulosa* and the three *Pontederia* species exhibited bands at 1580–1602 cm^−1^ and 1514–1539 cm^−1^, attributed to C=C stretching of aromatic rings and phenolic skeletal vibrations. These bands are consistent with aromatic and phenolic-related functional groups potentially associated with secondary metabolites. *P. stratiotes* and *T. geniculata* exhibited an intense and sharp band at 1740 cm^−1^, corresponding to C=O stretching of esters and fatty acids. This band was not detected in the other extracts and is consistent with ester carbonyl-containing compounds. These results suggest distinct chemical profiles obtained with methanol.

Overall, aqueous extracts exhibited relatively similar FTIR profiles, characterized by prominent O–H and C–O absorption bands. Methanolic extracts showed greater spectral variation among species, particularly in regions associated with aromatic, phenolic-related, and ester carbonyl functional groups. The original FTIR spectra of all aqueous and methanolic extracts for each species are provided in the Supplementary Material (Figures S3–S8).

### 2.4. Biostimulant Activity Assay

The two-way ANOVA revealed significant effects for a wide range of variables. Table 4 summarizes the ANOVA results for all bioassay variables. Treatment effects were significant for all variables, with highly significant *p*-values (*p* < 0.001) for most growth-related variables. Extraction effects were significant only for cotyledon dry mass, root dry mass, and the calculated ratios. Significant interactions between treatment and extraction were observed for epicotyl dry mass, cotyledon dry mass, root dry mass, shoot and root length, and the Root/Epicotyl ratio. Germination percentage, Germination Speed Index, and the Root/Shoot ratio showed no significant extraction effect or interaction.

The highest coefficients of variation were observed for root and shoot lengths, whereas the lowest coefficients were recorded for germination percentage, Germination Speed Index, and epicotyl and cotyledon dry mass. The behavior of the coefficients of variation was consistent with what is expected for experiments of this nature, which is consistent with the expected variability for this type of experiment. Means and standard errors are presented in bar graphs and detailed in the following sections.

The germination variable graphs (percentage and speed index) are presented in Figure 1. The significant treatment effect observed for Germination Speed Index (*p* < 0.01; Figure 1B) was driven by differences within the methanolic extract, where the treatments were divided into two groups: the lowest values corresponded to extracts from *Eleocharis cellulosa* and *Pontederia* sp., while extracts from all other species did not differ statistically from the control. Both species showed a GSI of 10.0, which was 0.82 points below the overall mean. The difference compared to the water + Tween control was 0.71 points.

Overall means for germination percentage (Figure 1A) were 92.6% and 92.4% for aqueous and methanolic extracts, respectively (Table 4). Although no significant differences among treatments were detected by the Scott-Knott test, the lowest germination value in the aqueous extract was 87% for *P. stratiotes*, while the highest was 97% for *P. rotundifolia*. In the methanolic extract, values ranged from 86% (*E. cellulosa*) to 96% (*P. azurea* and *T. geniculata*).

These data indicate that the extracts did not markedly affect final germination percentage, possibly due to the high physiological quality of the seeds used. The methanolic extract of two of the tested species reduced the germination speed in soybean, a response that should be interpreted with caution but will be considered in the overall discussion of the data.

Epicotyl dry mass (shoot without cotyledons) differed among species, with a significant treatment × extraction interaction (*p* < 0.01; Figure 2A). In the aqueous extract, two distinct significance groups were formed: the group with higher values included *T. geniculata* (0.38 g), *P. rotundifolia* (0.39 g), and *P. azurea* (0.39 g), while the group with lower values comprised the other three species and the control. The difference between the highest values and the control ranged from 0.04 to 0.05 g.

In the methanolic extract, in contrast, four significance groups were formed (Figure 2A). The highest value was observed for *P. azurea* (0.44 g), differing by 0.08 g from the control (0.36 g). The second highest value was recorded for *T. geniculata* (0.39 g), which is 0.03 g above the control. The third statistical group included the control and *P. stratiotes*, with means of 0.36 and 0.37 g, respectively. The last group, with the lowest values, comprised *P. rotundifolia*, *Pontederia* sp., and *E. cellulosa*, with masses ranging from 0.31 to 0.34 g.

Cotyledon dry mass showed significant treatment, extraction, and interaction effects (*p* < 0.01, *p* < 0.01, and *p* < 0.001, respectively; Figure 2B). In the aqueous extract, means were separated into three statistical groups: *E. cellulosa* (2.30 g), *P. stratiotes* (2.37 g), and *P. rotundifolia* (2.29 g) produced cotyledons heavier than the control (2.13 g). *T. geniculata* (2.11 g) was statistically equal to the control, whereas *Pontederia* sp. and *P. azurea* showed the lowest cotyledon dry mass values. In the methanolic extract, values ranged from 1.89 to 2.17 g, with an overall mean of 2.01 g, and no group differed from the control.

Root dry mass differed among species, with significant treatment, extraction, and interaction effects (*p* < 0.0001; Figure 2C). In the aqueous extract, two statistical groups were formed, with *E. cellulosa* (0.34 g), *P. azurea* (0.32 g), and *P. rotundifolia* (0.30 g) composing the group with higher values, while the other three species remained statistically equal to the control. Root dry mass in the second group ranged from 0.23 g (*Pontederia* sp.) to 0.27 g (control). The overall mean of root dry mass in the aqueous extract was 0.28 g.

In the methanolic extract, two statistical groups were also formed, but with a different pattern. The group with higher values included *T. geniculata*, *P. azurea*, *P. stratiotes*, *P. rotundifolia*, and the control, with mass ranging from 0.25 g (control) to 0.29 g (*T. geniculata*). The extracts of *E. cellulosa* and *Pontederia* sp. shared the group with the lowest root dry mass, both at 0.21 g. Notably, the control was grouped with the higher values in the methanolic extract, in contrast to its position in the aqueous extract.

The biomass variables showed a significant treatment × extraction interaction (Figure 2), indicating that the response of each species depended on the extraction method and that the effect was organ-specific. For epicotyl dry mass, *P. azurea* was the only species with a significant difference between extracts, with higher values in the methanolic extract. For cotyledon dry mass, *E. cellulosa* showed the opposite pattern, with higher values in the aqueous extract. For root dry mass, three species showed significant differences between extracts: *E. cellulosa* and *P. azurea* had higher values in the aqueous extract, whereas *T. geniculata* had higher values in the methanolic extract. These organ-specific responses indicate that the effect of the extracts on biomass allocation was not uniform across species, organs, or extraction methods.

Shoot and root lengths were significantly affected by treatment and by the treatment × extraction interaction (Table 4). In the aqueous extract, three statistical groups were formed for shoot length (Figure 3A). The highest values were observed for *T. geniculata* (9.27 cm) and *P. azurea* (8.96 cm), forming a group statistically distinct from the control (8.29 cm). The intermediate group included *P. rotundifolia* (8.79 cm), *E. cellulosa* (8.48 cm), and the control. The lowest values were observed for *Pontederia* sp. (8.08 cm) and *P. stratiotes* (7.58 cm).

In the methanolic extract, in contrast, three statistical groups were observed for shoot length (Figure 3A). The extract of *P. rotundifolia* stood out as the highest isolated value at 9.79 cm, an increase of 0.70 cm compared to the control (9.09 cm). The extracts of *P. stratiotes* and *T. geniculata* were statistically equal to the control, with values ranging from 9.04 cm (*T. geniculata*) to 9.09 cm (control). The extracts of *E. cellulosa* (7.66 cm), *Pontederia* sp. (7.75 cm), and *P. azurea* (7.83 cm) were statistically lower than the control.

Root length was significantly greater in seedlings treated with aqueous extracts of *E. cellulosa*, *T. geniculata*, and *P. rotundifolia*, with values of 13.37, 13.65, and 13.47 cm, respectively, and increases of up to 1.21 cm compared to the control (12.44 cm; Figure 3B). *P. azurea* and *P. stratiotes* formed the intermediate group, statistically equal to the control. The shortest root length occurred in seedlings treated with *Pontederia* sp. extract, with a difference of 1.16 cm compared to the control and 2.19 cm compared to *P. rotundifolia*.

In the methanolic extract, in contrast, the treatments were separated into three distinct groups (Figure 3B). The extracts of *P. rotundifolia* (14.62 cm), *T. geniculata* (13.95 cm), and *P. stratiotes* (13.95 cm) were statistically greater than the control (12.58 cm). A difference of 2.04 cm was noted between the control and the highest recorded value. On the other hand, roots were markedly shorter in the extracts of *P. azurea* (11.00 cm), *E. cellulosa* (10.74 cm), and *Pontederia* sp. (10.71 cm), with a difference of 3.91 cm between the highest and the lowest value.

Root/Epicotyl ratio showed significant treatment, extraction, and interaction effects, whereas Root/Shoot ratio showed a significant treatment effect only (Table 4, Figure 4).

The length variables showed a significant treatment × extraction interaction (Table 4), indicating that the effect of each species on shoot and root elongation depended on the extraction method and was organ-specific. For shoot length, some species performed better in the aqueous extract, while others performed better in the methanolic extract. The same organ-specific pattern was observed for root length, with the direction of the response varying among species. *T. geniculata* was the only species without a significant difference between extracts for either variable. These organ-specific responses indicate that the extracts affected shoot and root elongation in different ways depending on the species and the extraction method.

Values of Root/Epicotyl ratio ranged from 0.69 to 0.99 (aqueous) and from 0.64 to 0.76 (methanolic). Root/Shoot ratio ranged from 1.45 to 1.65 (aqueous) and from 1.42 to 1.57 (methanolic). For the Root/Epicotyl ratio, the significant interaction reflected contrasting responses between extracts in *E. cellulosa*, *P. stratiotes*, and *P. azurea*. These patterns are consistent with the absolute growth data discussed earlier.

In summary, the bioassay results revealed significant treatment effects across variables, with organ-specific responses depending on the extraction method. Germination percentage showed no significant separation among treatments by the Scott-Knott test (Figure 1A), while Germination Speed Index showed a significant treatment effect, with the lowest values observed in the methanolic extracts of *E. cellulosa* and *Pontederia* sp. (Figure 1B). Among biomass and length variables, *P. rotundifolia*, *T. geniculata*, and *P. azurea* were among the highest values in several cases, whereas *E. cellulosa* and *Pontederia* sp. were among the lowest (Figure 2A,B and Figure 3A,B). Notably, *P. stratiotes* showed intermediate values in most variables, standing out only for root length in the methanolic extract (Figure 3B). A significant treatment × extraction interaction was observed for most growth variables, indicating that the response of each species depended on the extraction method.

## 3. Discussion

### 3.1. Environmental Context and Extraction Efficiency from Field to Extract

The macrophytes were collected in early November 2025, during an exceptionally dry period in the Pantanal, which provides context for the interpretation of the results. Maximum temperatures ranged from 33 °C to 37 °C, and relative humidity dropped to critical levels between 15% and 30% during the hottest hours. No prior rainfall had been recorded in the Nhecolândia sub-region. The first half of November 2025 registered the lowest rainfall index in the entire state of Mato Grosso do Sul for the Pantanal region, with accumulated precipitation well below the historical average [21]. During the field expedition, a single rainfall event occurred, followed by another dry period. At the end of November, the rains returned and marked the beginning of the flood season.

These field conditions are relevant for interpreting the chemical profile of the extracts. Under combined heat and water stress, aquatic macrophytes tend to accumulate secondary metabolites such as phenolic compounds, flavonoids, alkaloids, and terpenoids, which act as osmoprotectants and antioxidants [22]. When extracted and applied to other plants, these same compounds can have either stimulatory or inhibitory effects, depending on their concentration and chemical nature [23]. The contrasting responses observed even among closely related *Pontederia* species, collected from ponds at slightly different stages of drying, suggest that local microhabitat variation may influence the intensity of stress experienced by each plant and, in turn, the bioactivity of its extract, although this was not directly measured in this study. It is important to note that the environmental conditions described here provide context for the study; they were not manipulated as experimental variables.

The FTIR profiles are consistent with divergent metabolic strategies among the studied species [24,25,26]. A possible interpretation, consistent with the literature, is that *Pontederia* and *Eleocharis* may accumulate protective phenolic compounds (bands at ~1514–1602 cm^−1^), whereas *Pistia* and *Thalia* may prioritize esters and lipid cuticular waxes (band at 1740 cm^−1^). Although these interpretations are supported by the literature, they were not directly tested in this study.

The divergent metabolic plasticity observed, with *Pontederia* favoring phenolics and *Pistia* investing in cuticular waxes, may reflect adaptive strategies to synergistic abiotic stressors such as high UV radiation and temperature [27]. This differential resource allocation is supported by the Protein Competition Model, which predicts an increase in secondary metabolites when environmental stress limits primary growth [28,29,30]. Collecting under real field conditions, as done here, provides a snapshot of the phenological state of these species at a specific moment, which is relevant for future valorization of their biomass [31]. The significant treatment × extraction interactions observed for most variables (Table 4) support the idea that the chemical composition of each extract and not only the species identity drives the biological responses.

Regarding processing, total dry biomass varied considerably among species, ranging from 1.22 kg (*Pontederia* sp.) to 6.66 kg (*Thalia geniculata*), reflecting both morphological differences and material availability at each collection point. The milling yield was approximately 91.4%, a value consistent with those reported for this type of processing [32,33].

Regarding extraction efficiency, the inverse relationship between yield and retention confirmed that the mass balance was adequately controlled. *Pontederia rotundifolia* showed the highest aqueous yield (21.37%) and the lowest retention (67.50%), indicating high solubility of its polar components. In contrast, the methanolic extract of *Eleocharis cellulosa* had the lowest yield (4.84%) and the highest retention (90.05%), suggesting a biomass rich in insoluble fibers. Species with higher yields tended to show greater bioactivity, but the correspondence was not perfect: *Pistia stratiotes* had a high aqueous yield (11.70%) yet did not stand out in bioactivity, reinforcing that the chemical composition of what is extracted matters more than the absolute amount [34].

The extraction yields observed in this study, such as the 21.37% obtained for *Pontederia rotundifolia*, are strongly supported by the literature, which reports similar values for aqueous and hydroalcoholic extracts of macrophytes and medicinal plants, typically ranging from 22.17% to 29.75% [35,36]. This variability, together with the 8.6% processing loss, confirms that mass balance efficiency was achieved. It also reinforces the premise that chemical composition outweighs the absolute amount extracted in determining bioactivity.

In the specific case of *Pistia stratiotes*, which did not promote soybean growth despite its high extraction yield, one hypothesis is that the extract of this species shows potential inhibitory activity against microorganisms in other biological models [36]. In the literature, the chemical profile of *Pistia* has been associated primarily with inhibitory and defensive roles, which may explain why its high solubility results in inhibitory effects in other models [37,38], but did not translate into physiological stimulation of soybean germination in the present study. Although this hypothesis is supported by the literature, it was not directly tested here. These observations reinforce the distinct behavior of *Pistia* across biological models.

### 3.2. Germination and Seedling Growth Responses

The extracts did not inhibit final germination at the tested concentrations, since germination percentage did not differ significantly among treatments [39,40,41]. Overall means were 92.6% for aqueous extracts and 92.4% for methanolic extracts. In the two-way ANOVA, germination percentage showed a significant treatment effect (*p* = 0.0046), but the Scott-Knott test did not separate the means (Table 4). These results suggest that the extracts did not impair the completion of germination under the tested conditions, although this does not imply absence of other physiological effects [42,43,44].

Although final germination was not affected, the extracts influenced germination speed in two specific cases. The germination speed index was reduced by the methanolic extracts of *E. cellulosa* and *Pontederia* sp., both with an absolute value of 10.0, 0.82 points below the overall mean and 0.71 points below the water + Tween control. In the two-way ANOVA, GSI showed a significant treatment effect (*p* = 0.0085), with no significant extraction effect or interaction (Table 4). This delay may be related to the presence of phenolic compounds and other allelochemicals, which, depending on concentration and extraction method, can slow water imbibition or initial enzymatic activation [44,45]. In the allelopathy literature, substances such as simple phenols and organic acids have been reported to change germination speed without necessarily reducing the total number of germinated seeds [46,47].

The seed response to crude extracts may depend on a complex balance between stimulatory and inhibitory substances. Phytohormones and precursors present in algae and macrophytes, such as auxin, cytokinin, and gibberellin-like substances, regulate developmental processes [47,48]. Plant extracts can also induce amylase activity independently of gibberellin, speeding up the mobilization of reserves to the embryo, while betaines and polysaccharides help maintain cellular integrity and water balance during germination [49,50]. The application of extracts rich in elicitors, such as polysaccharides, may cause the seed to divert metabolic energy toward the activation of defense pathways before completing emergence, resulting in a subtle physiological delay [51,52,53]. In summary, the combination of unchanged final germination with reduced GSI indicates that some extracts affected the rate, but not the final completion, of germination. Whether this response is related to defense elicitation or other metabolic modulation requires further physiological and biochemical investigation, without compromising basic viability.

Regarding seedling growth, *P. rotundifolia* and *T. geniculata* showed the most consistent positive effects across growth variables, whereas the response to *P. azurea* depended on extract type and growth parameter, as indicated by the significant treatment × extraction interaction (Table 4). These responses may be related to the presence of bioactive compounds that stimulate natural plant processes [48,54,55]. In the literature, the main driver of early radicle and hypocotyl growth is the action of phytohormones, especially auxins [10,56]. Phenolic acids such as ferulic acid have been reported to protect indole-3-acetic acid (IAA) from enzymatic degradation [57], maintaining higher levels of active auxin in the seedling and promoting both the number of adventitious roots and root elongation. Certain phenolic compounds, such as eckol and phloroglucinol, exhibit auxin-like activity, directly stimulating root development shortly after radicle emergence [58,59,60]. Although these mechanisms are supported by the literature, they were not directly measured in this study.

Seed treatment with the extracts may have acted as biological elicitors, in which secondary metabolites present in the crude extracts could accelerate seed metabolism [51]. Flavonoids and phenolic acids may act as antioxidants, protecting young tissues against oxidative damage, while the balance of these phenols could increase the activity of enzymes such as superoxide dismutase (SOD) and catalase (CAT), contributing to cellular stability during tissue expansion [52]. Plant-derived extracts can contain compounds capable of modulating hormonal signaling, antioxidant metabolism, and early plant development [48,51,52,53,54,55,56,57,58,59,60]. Such mechanisms may contribute to the growth responses observed in this study; however, neither phytohormones nor antioxidant enzyme activities were directly measured.

In contrast, *E. cellulosa* and *Pontederia* sp. showed neutral or negative effects on most growth variables. These null or negative effects may be related to allelopathic mechanisms: some plants produce allelochemicals such as terpenoids, alkaloids, and certain phenolics that, in other systems, have been associated with tissue disorganization and the accumulation of reactive oxygen species (ROS) in treated plants [44,61,62,63,64]. Moreover, the effect of an extract is critically dose-dependent: very low concentrations may be ineffective due to excessive dilution, while very high doses may become phytotoxic [49].

The variation relative to the control, with some species outperforming it and others falling below, is a common feature of crude extracts, which are complex mixtures of hundreds of molecules [65,66]. The biostimulant effect is likely to occur when the beneficial compounds outweigh the potential inhibitors present in the same sample. In summary, based on the screening conditions tested here, *P. rotundifolia*, *T. geniculata*, and *P. azurea* showed promising biostimulant potential, whereas *E. cellulosa* and *Pontederia* sp. did not show this potential.

### 3.3. FTIR Profiles and Comparison with Other Macrophytes

FTIR analysis revealed distinct functional group profiles between aqueous and methanolic extracts. The aqueous extracts of all species showed a broad O–H stretching band between 3254 and 3281 cm^−1^ and strong C–O–C bands in the region from 1007 to 1072 cm^−1^, consistent with the presence of polysaccharides [67,68]. These compounds are frequently associated with biostimulant activity in seaweed extracts [67,69].

In the methanolic extracts, more intense C–H stretching bands were observed at 2921–2923 and 2850–2851 cm^−1^, suggesting the presence of aliphatic chains from lipids and fatty acids [70]. The presence of aromatic C=C bands between 1580 and 1605 cm^−1^ and between 1514 and 1539 cm^−1^ suggested the preferential extraction of flavonoids and phenolic compounds by methanol [71].

The extracts of *E. cellulosa* and *Pontederia* sp., which showed neutral or negative effects on most growth variables, also showed strong aromatic bands in the methanolic extracts (1580 and 1514 cm^−1^ in *E. cellulosa*; 1589 cm^−1^ in *Pontederia* sp.). These bands are consistent with the presence of flavonoids and phenolic acids [64], which is consistent with the hypothesis that these compounds may contribute to the observed inhibitory effects. In *Eichhornia crassipes*, FTIR bands have been associated with alkaloids and phthalate derivatives showing antimicrobial and antialgal activity [71,72]. The presence of an aromatic band in the aqueous extract of *Pontederia* sp. (1590 cm^−1^) may also help explain why this species showed an inhibitory tendency even in the more polar extract.

In contrast, *T. geniculata*, *P. rotundifolia*, and *P. azurea*, the species with the most consistent positive responses in the bioassay, showed richer and more balanced spectra. *P. rotundifolia* and *P. azurea* combined polysaccharide bands (~1063 and 1072 cm^−1^, respectively) with moderate aromatic bands (~1587 and 1597 cm^−1^), suggesting a favorable balance between stimulatory and antioxidant compounds [67,69,73]. *T. geniculata* stood out as one of only two species to show the ester C=O band at 1740 cm^−1^ in the methanolic extract, together with *P. stratiotes*. However, only *T. geniculata* showed consistent positive effects, while *P. stratiotes* did not differ from the control. This divergence indicates that the simple presence of a compound class does not determine the biological effect, specific molecular identity and concentration are equally decisive [64,67]. The esters in *Thalia* may represent lipophilic hormone precursors or bioactive fatty acids although this was not directly tested [73], whereas those in *Pistia* may be structurally distinct or present at insufficient concentrations.

*P. azurea* deserves special mention: its methanolic extract showed the most intense aromatic band among all species (1539 cm^−1^), with a pronounced aromatic character, while also retaining a polysaccharide band (1028 cm^−1^). This dual profile is consistent with the bimodal behavior observed in the bioassay, the highest epicotyl dry mass (0.44 g) combined with low root length (11.00 cm). The contrasting responses observed for different growth variables in the *P. azurea* methanolic extract may reflect the complex composition of the crude extract. However, identification and quantification of the compounds involved will be necessary to explain these organ-specific responses [64,68,69].

When comparing our results with the literature on other aquatic macrophytes, parallels and contrasts emerge. *Lemna minor* is the most studied freshwater macrophyte as a biostimulant: aqueous extracts of this species improved net photosynthesis and chlorophyll content in olive trees, stimulated photosystems in tomato plants, and promoted maize growth [74,75,76]. The polysaccharide profile observed in *T. geniculata* and *P. rotundifolia* is comparable to that described for *Lemna*, suggesting that biostimulant potential is not restricted to a single genus or botanical family.

*Eichhornia crassipes*, on the other hand, is documented for its allelopathic effects mediated by phenolic compounds, which reduce chlorophyll content and induce lipid peroxidation in recipient plants [64,72]. The aromatic bands detected in *E. cellulosa* and *Pontederia* sp. align with this profile, reinforcing that freshwater macrophytes are sources of both stimulatory and inhibitory compounds.

Our data expand this knowledge to species never before characterized for this purpose. *Thalia geniculata*, *Pontederia rotundifolia* and *Pontederia azurea* are presented here for the first time as potential sources of biostimulants, with FTIR profiles that bring them closer to *Lemna minor* in terms of chemical classes. *Eleocharis cellulosa*, equally unprecedented, aligns with the allelopathic pattern of Cyperaceae and *Eichhornia*. The variation observed even among three congeneric *Pontederia* species, one stimulatory, one inhibitory, and one with a mixed response, suggests that phylogeny alone does not predict bioactivity. Microhabitat, collection conditions, and the specific chemical composition of each species are equally determinant.

It is important to recognize that FTIR identifies functional groups, not specific compounds. Bands in the same region may originate from different molecules, and relative intensity does not reflect absolute concentration without careful normalization. The definitive attribution of bioactivity to individual compounds will require complementary techniques such as GC-MS or LC-MS. Nevertheless, FTIR provided an objective basis for interpreting the biological data and for generating preliminary hypotheses about the compound classes potentially contributing to the observed effects.

### 3.4. Implications, Limitations, and Future Directions

The results of this study have direct implications for the use of Pantanal macrophytes as a source of agricultural bioinputs. The species *P. rotundifolia*, *T. geniculata*, and *P. azurea* showed potential as biostimulants for soybean, with positive effects on biomass and length in most cases and no negative effect on final germination. These three species are abundant in the Pantanal, and *T. geniculata* had the highest dry biomass yield (6.66 kg), which is relevant for scaling feasibility. On the other hand, *E. cellulosa* and *Pontederia* sp., which showed neutral or negative effects on most growth variables, may warrant further investigation as sources of compounds with potential inhibitory activity, for future applications as biocides or bioherbicides, diversifying the portfolio of potential bioinputs from the biome.

The significant treatment × extraction interactions observed for most growth variables (Table 4) reinforce that the response to each extract depends on both the species and the extraction method, which has direct implications for the selection and formulation of future bioinputs.

The fact that aqueous extracts produced positive biological responses reinforces the sustainability of this approach, since water is a low-cost, low-toxicity, and widely available solvent, compatible with the principles of green chemistry. The use of these macrophytes as bioinputs aligns with the principles of circular bioeconomy [77,78]. Species such as water hyacinth and water lettuce are often treated as invaders that block canals and drainages; harvesting them to obtain biostimulant or inhibitory extracts could transform an environmental problem into an economic resource [13,73,79].

This work presents contributions on three scales. Locally, it is the first to evaluate the biostimulant potential of aquatic macrophytes from the Pantanal, generating scientific knowledge on species never before tested for this purpose. Regionally, it places the Pantanal on the bioprospecting map for bioinputs, a field dominated by studies on marine algae and terrestrial plants, and may encourage studies in other Brazilian biomes equally rich in macrophytes, such as the Amazon and the Cerrado. Globally, the work contributes to filling a relevant gap in the literature: the scarcity of data on continental freshwater macrophytes as sources of agricultural bioinputs, in contrast with the vast scientific production on marine seaweed extracts [13]. In doing so, it presents original results and provides a methodological and conceptual basis that may serve as a starting point for similar initiatives in other regions of the world where aquatic macrophytes are abundant and underutilized.

The scope of this study reflects its position as an early-stage bioprospecting effort, consistent with the low technological readiness level (TRL) of this research line. The use of a single model crop (soybean), a single concentration per extract, and a single collection expedition limits the generalization of the results. Chemical characterization was based on FTIR, which identifies functional groups rather than specific compounds; complementary techniques such as GC-MS or LC-MS and field validation remain future steps. Given that continental freshwater macrophytes are still a largely unexplored niche for bioinput development, these boundaries are consistent with the intended scope and provide a foundation for subsequent studies.

## 4. Material and Methods

### 4.1. Collection of Aquatic Plant Samples

An expedition was conducted in *Nhecolândia*, a sandy sub-region of the Pantanal of “Mato Grosso do Sul” state, to collect aquatic plants from lotic and lentic environments, including rivers and shallow subcircular lakes. Six collection points were georeferenced for sampling (Figure 5). Access to the Brazilian genetic heritage was registered in SISGEN under registration number AA6354A.

Plant specimens were manually collected from their natural habitats, thoroughly washed to remove sediment, debris, and epiphytic organisms, and placed in sealed, properly labeled plastic bags for transport to the laboratory.

### 4.2. Processing of Collected Plant Material

A portion of each specimen was separated and preserved for molecular identification and phylogenetic analyses, enabling accurate taxonomic characterization of the collected species. The remaining plant material was subjected to a preliminary drying process under natural sunlight on benches lined with kraft paper, followed by oven drying in a forced-air circulation oven at 45 °C until a constant weight was achieved. Subsequently, the dried material was ground using a knife mill (model SL32, Solab, Piracicaba, Brazil) fitted with a 30-mesh sieve to obtain a homogeneous powder. The residual moisture content of the milled biomass stored for extractions was determined using a thermogravimetric halogen moisture analyzer balance at 105 °C until a constant weight was reached. The processed samples were then aliquoted into appropriate storage bags and maintained at −20 °C until further analyses.

### 4.3. Morphological and Molecular Identification of Plant Species

#### 4.3.1. DNA Extraction

Plant identification was initially performed based on morphological characteristics, followed by molecular confirmation. Genomic DNA was extracted from fresh leaf, with quality and concentration evaluated prior to analysis. Approximately 50 mg of plant biomass was used for genomic DNA extraction using the QIAamp DNA Kit (Qiagen, Venlo, The Netherlands), following the manufacturer’s instructions. DNA concentration and purity were determined using a NanoDrop 2000 spectrophotometer by Thermo Fisher Scientific (Waltham, MA, USA).

#### 4.3.2. PCR Amplification

PCR amplification was performed in a Veriti Thermal Cycler (Thermo Fisher Scientific) using the GoTaq^®^ G2 DNA Polymerase Kit (Promega, Madison, WI, USA), according to the manufacturer’s recommendations.

Each reaction contained 20 ng of purified genomic DNA. For plant identification, a partial region of the ribulose-1,5-bisphosphate carboxylase/oxygenase large subunit (*rbcL*) gene was amplified using the primers rbcLa-F (5′-ATGTCACCACAAACAGAGACTAAAGC-3′) and rbcLa-R (5′-GTAAAATCAAGTCCACCRCG-3′) [80]. Thermal cycling conditions consisted of an initial denaturation at 94 °C for 5 min, followed by 35 cycles of denaturation at 94 °C for 30 s, primer annealing at 55 °C for 30 s, and extension at 72 °C for 45 s, with a final extension at 72 °C for 7 min. PCR products were analyzed by electrophoresis on 1% agarose gels prepared in TAE buffer. Amplicons were subsequently purified using Exonuclease I (Exo I) and Shrimp Alkaline Phosphatase (SAP) (Thermo Fisher Scientific), following the manufacturer’s instructions.

#### 4.3.3. Sanger Sequencing

Purified PCR products were sequenced using the BigDye^®^ Terminator v3.1 Cycle Sequencing Kit (Thermo Fisher Scientific), according to the manufacturer’s recommendations. Sequencing reactions contained 50 ng of purified PCR product and were subjected to 35 cycles consisting of denaturation at 96 °C for 15 s, primer annealing at 60 °C for 15 s, and extension at 60 °C for 4 min. Sequencing products were purified by precipitation with 20% ammonium acetate (7.5 M) and three volumes of absolute ethanol, resuspended in 10 μL of Hi-Di™ Formamide, and analyzed on an ABI 3500xL Genetic Analyzer (Thermo Fisher Scientific) equipped with 50 cm capillaries and POP-7 polymer, according to the manufacturer’s recommendations.

#### 4.3.4. Bioinformatics Analysis

Chromatograms were processed using Sequencing Analysis Software v5.4 (Thermo Fisher Scientific). Sequence quality was assessed using a Phred quality score threshold greater than 25. Forward and reverse reads were assembled into consensus sequences using CAP3 software. Consensus sequences were compared against the NCBI non-redundant (nr) nucleotide database using BLASTn for taxonomic identification. These identifications rely on comprehensive and accurate reference libraries of standard genetic markers, such as the *rbcL* library developed in this study. Of the six aquatic plant species analyzed, molecular identification was successfully achieved for five specimens (Table 5).

Partial sequencing of the chloroplast rbcL gene yielded high-quality amplicons ranging from 701 to 730 bp for five aquatic macrophyte species. BLASTn analysis against the NCBI non-redundant nucleotide database confirmed 99.0% sequence identity across all molecularly identified species: *Thalia geniculata* (701/702 bp, 99% identity), *Eleocharis cellulosa* (727/730 bp, 99% identity), *Pontederia* sp. (715/716 bp, 99% identity), *Pontederia azurea* (716/718 bp, 99% identity), and *Pontederia rotundifolia* (716/718 bp, 99% identity).

The sequences generated in this study were deposited in GenBank, and the corresponding accession numbers are provided as follows: *Thalia geniculata* (QB046331), *Pontederia* sp. (QB046333), *Eleocharis cellulosa* (QB046332, QB046334, QB046336), *Pontederia azurea* (QB046335), and *Pontederia rotundifolia* (QB046337). Species collected at more than one sampling point yielded more than one sequence, and all corresponding accession numbers are listed.

Molecular identification could not be achieved for *Pistia stratiotes* due to unsuccessful DNA amplification during PCR reaction, possibly related to the delicate and highly hydrated nature of its tissues, which may compromise DNA integrity after field collection. Therefore, taxonomic identification was based on morphological characters using appropriate botanical criteria [81].

### 4.4. Preparation of Aqueous and Methanolic Extracts

Previously dried and ground samples of Pantanal aquatic plants were granulometrically standardized using a 30-mesh sieve. The standardized biomass was subjected to dynamic maceration in Erlenmeyer flasks containing 100 mL of extraction solvent under orbital agitation (Multitron Standard, INFORS^®^ HT, Bottmingen, Switzerland) at 200 rpm for 24 h at 25 °C. Three stainless-steel beads were added to each flask to improve homogenization during extraction.

For methanolic extraction, 10g of dried plant material were used for all samples. For aqueous extraction, the amount of biomass was adjusted according to the water absorption capacity of each species. Samples exhibiting high water retention, which could compromise homogenization and extraction efficiency, were processed using reduced biomass quantities: *Pontederia azurea* (8 g); *Pontederia rotundifolia* (5 g) and, *Pontederia* sp. (5 g).

Different separation procedures were applied according to the solvent used. Methanolic extracts were filtered through pre-dried and pre-weighed quantitative filter paper. Aqueous extracts were initially filtered through voile fabric, and the resulting filtrates were centrifuged at 10.000 rpm and 4 °C for 10 min to remove fine suspended particles.

The solid residues retained during filtration were dried in a forced-air oven until constant weight and subsequently weighed to determine retained biomass. Methanolic filtrates were concentrated under reduced pressure using a rotary evaporator (model TE-211, Tecnal^®^, Piracicaba, SP, Brazil) at 45 °C, followed by final solvent removal in a fume hood and drying in a forced-air oven at 40 °C until constant weight. Aqueous extracts were dried directly in a forced-air oven at 40 °C until complete water evaporation and constant weight were achieved. The resulting crude extracts were transferred to sterile Petri dishes, sealed with laboratory film, and stored at −20 °C until further analysis [82].

### 4.5. Determination of Extraction Yield and Retention

Extraction yield on a constant dry weight basis was calculated as the ratio between the dry extract weight and the initial dry biomass weight, retention was determined as the ratio between the dry weight of retained solid residue and the initial dry biomass weight, and the total balance was determined as the sum of the biomass of the extract and residue [83]. All extractions were performed in triplicate, and results were expressed as mean ± sample standard deviation (SD).

### 4.6. Fourier Transform Infrared (FTIR) Spectroscopic Characterization

Fourier Transform Infrared spectroscopy was performed to characterize the chemical composition of the plant extracts by identifying the main functional groups present in their molecular structures. ATR-FTIR spectra were recorded using a Nicolet iS10 spectrometer (Thermo Fisher Scientific, Waltham, MA, USA) equipped with a single-reflection ATR accessory with a diamond crystal (45° incidence angle). Spectra were acquired in the 4000–400 cm^−1^ range at 4 cm^−1^ resolution with 32 co-added scans at room temperature. A background spectrum was collected under the same conditions prior to each run. All spectra were baseline-corrected and min–max vector normalized using OMNIC™ Software (v9.2). Three independent extract preparations (*n* = 3) were analyzed per treatment.

The obtained spectra were analyzed based on the characteristic vibrational bands of chemical bonds, allowing the identification of functional groups associated with primary and secondary plant metabolites. FTIR spectroscopy is a rapid, non-destructive, and reliable analytical technique widely employed for the structural characterization of plant-derived extracts, providing valuable information on their chemical composition, molecular interactions, and sample purity [84].

### 4.7. Seed Treatment and Biostimulant Activity Assay

The biostimulant potential of aqueous and methanolic extracts obtained from Pantanal aquatic macrophytes were evaluated through seed treatment bioassays using soybean white seeds. Commercial soybean seeds (*Glycine max* L., cv. Brasmax Olimpo^®^, 2024/2025 harvest, initial germination > 90%) were standardized for size and health. Germination was recorded based on primary root protrusion (physiological criterion). The assays were conducted as a preliminary screening to identify extracts capable of enhancing seed physiological quality and promoting early seedling development.

Experimental procedures were conducted following the Brazilian Rules for Seed Analysis (RAS) established by the Ministry of Agriculture and Livestock, which constitute the official standard for evaluating seed physiological quality in Brazil [19]. The paper sheets were rolled and incubated in a BOD chamber (model 600-L INCF-5140-0062, Labor Stabille, Rome, Italy) maintained at 25 ± 2 °C, under a 12h light/12 h dark photoperiod throughout the evaluation period.

Seeds were treated with the extracts at a dose equivalent to 5 mL kg^−1^ of seeds. Prior to seed treatment, the aqueous extracts were diluted in distilled water and methanolic extracts were diluted in distilled water containing 0.25% (*v*/*v*) Tween 20 as a non-ionic surfactant to facilitate extract dispersion and ensure homogeneous seed coating. The final treatment solutions were prepared immediately before application. For laboratory-scale applications, 100 μL of each treatment solution were applied to 20 g of soybean seeds, ensuring uniform coverage of the seed surface. The concentrations of each extract are presented in Table 6.

Extract application concentrations were established based on a biomass-equivalent dose approach, where treatments reflect the natural extraction yield obtained from a standardized mass of dry starting material [67]. In early-stage bioprospecting of complex multicomponent bioinputs, evaluating crude extracts proportional to their raw biomass recovery provides a realistic assessment of biostimulatory efficiency per unit of harvested macrophyte, aligning with green extraction and circular bioeconomy principles [85].

The experiment was conducted in a randomized complete block design with four replicates per treatment, each consisting of 25 seeds. Following treatment, the seeds were distributed on Germitest^®^ paper towels moistened with distilled water at a volume corresponding to 2.5 times the dry weight of the paper [19]. Seedling parts (epicotyl, cotyledon, and root) were dried in a forced-air oven at 65 °C for 72 h until constant dry weight.

The following variables were evaluated: germination percentage (%), Germination Speed Index (GSI) [86], epicotyl dry mass (g), cotyledon dry mass (g), root dry mass (g), root/epicotyl ratio based on dry mass, shoot length (cm), root length (cm), and root/shoot ratio based on length.

### 4.8. Statistical Analysis

Data obtained from extraction yield were expressed as mean ± standard deviation (SD). The experiment was conducted using a randomized complete block design with four biological replicates per treatment and 25 seeds per replicate. Residual normality was verified using the Shapiro–Wilk test (*p* > 0.05) and homoscedasticity using Levene’s test (*p* > 0.05).

Data were subjected to two-way analysis of variance (ANOVA) to assess the effects of treatment (6 species and controls), extraction method (aqueous vs. methanolic) and their interaction. Germination percentage (G) and germination speed index (GSI) were determined over an 8-day evaluation period, with each replicate of 25 seeds constituting an experimental unit (*n* = 4). Epicotyl dry mass (EDM), cotyledon dry mass (CDM), root dry mass (RDM) and the root-to-epicotyl dry mass ratio (R/ER) were determined from pooled measurements of 25 seedlings per replicate, likewise considering the replicate as the experimental unit (*n* = 4). Shoot length (SL), root length (RL) and the root-to-shoot length ratio (R/SR) were measured individually in 25 seedlings per replicate, yielding 100 individual observations per treatment.

For inferential analyses, the randomized block structure and biological replication were retained to account for within-replicate dependence and avoid pseudoreplication. When ANOVA was significant, the Scott-Knott multiple comparison test was applied at a 5% significance level (*p* < 0.05). Graphs were generated using the ggplot2 package with patchwork for panel assembly. All statistical analyses were performed in R software version 4.5.2 and RStudio interface [87,88].

## 5. Conclusions

This study evaluated six aquatic macrophytes from the Pantanal as potential sources of agricultural bioinputs. None of the extracts inhibited final soybean germination at the tested concentrations. *Pontederia rotundifolia*, *Thalia geniculata*, and *Pontederia azurea* showed promising biostimulant potential under the tested conditions. *Eleocharis cellulosa* and *Pontederia* sp. showed neutral or negative effects on most growth variables.

The solvent influenced the chemical profile and the biological responses. Aqueous extracts showed bands consistent with polysaccharides and produced positive biological responses without organic solvents, an advantage for future scaling and sustainability. Methanolic extracts showed bands suggestive of flavonoids, esters, and lipids, with more variable effects. FTIR provided preliminary evidence for these interpretations: species with neutral or negative effects tended to show more intense aromatic bands, while species with positive effects exhibited a more balanced profile.

Overall, the results indicate that Pantanal macrophytes are a promising resource for further investigation as bioinputs, particularly through the aqueous extraction route. The findings provide a preliminary basis for future studies on dose–response, additional crops, and chemical characterization.

## Figures and Tables

**Figure 1:**
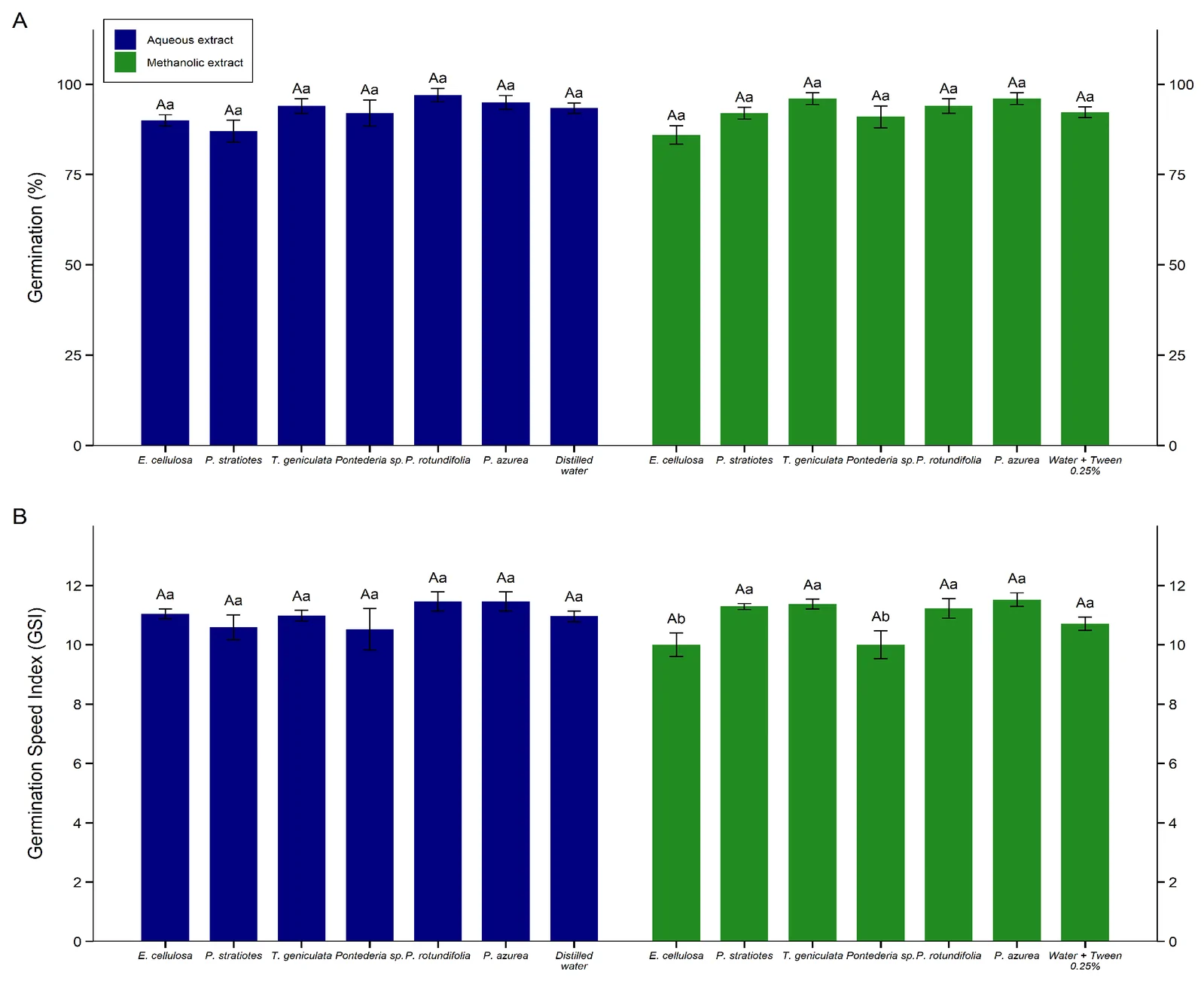
Germination percentage (**A**) and Germination Speed Index (**B**) of soybean seeds after treatment with aqueous and methanolic crude extracts obtained from six aquatic macrophyte species collected in the Pantanal biome. Distilled water and Tween 20 solution 0.25% (*v*/*v*) were used as controls for aqueous and methanolic extracts, respectively. Values are mean ± SE. Uppercase letters compare aqueous and methanolic extracts within each treatment; lowercase letters compare treatments within each extract. Means followed by the same letter do not differ by Scott-Knott test (*p* < 0.05).

**Figure 2:**
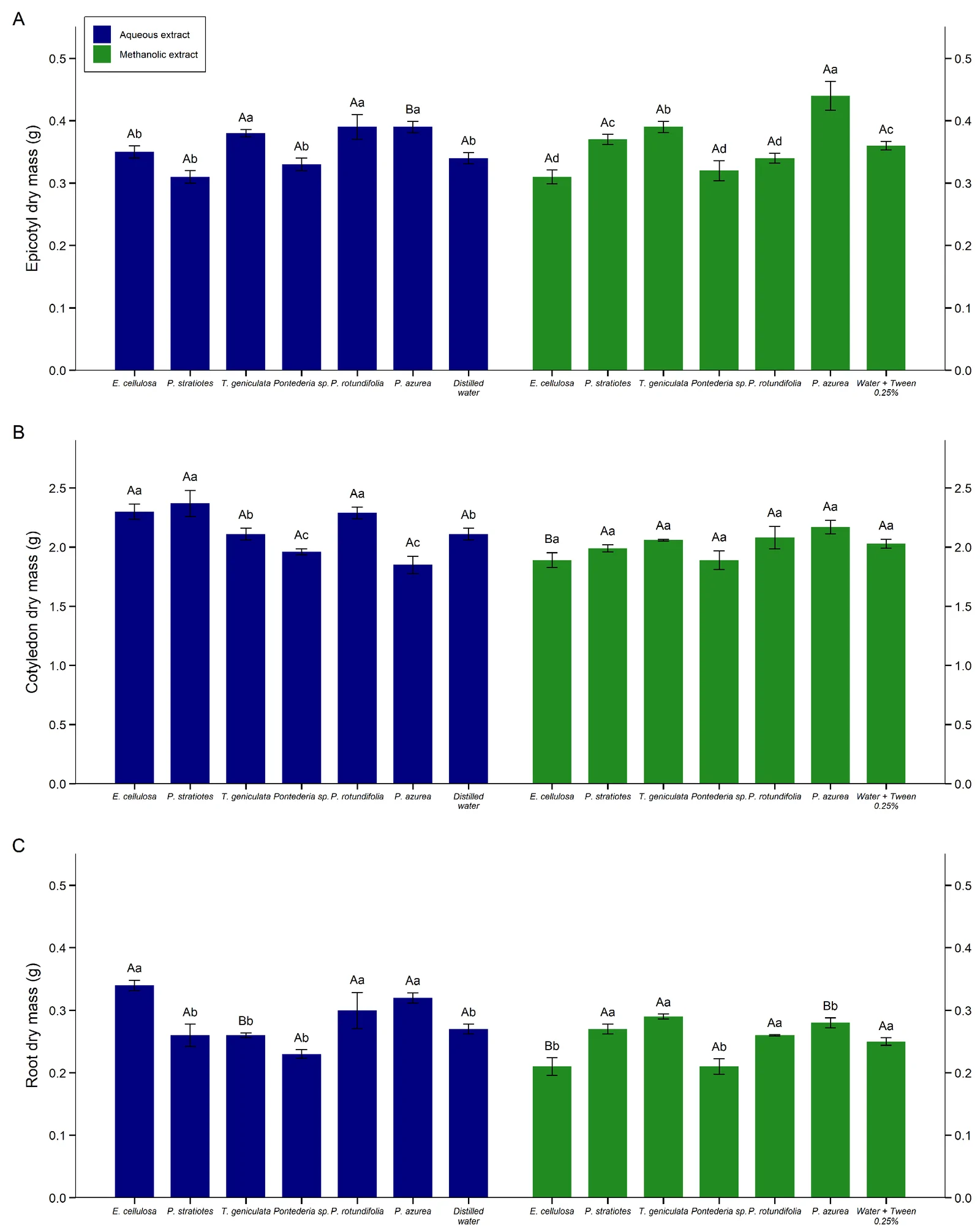
Epicotyl dry mass (**A**), cotyledon dry mass (**B**), and root dry mass (**C**) of soybean seedlings after treatment of seeds with aqueous and methanolic crude extracts obtained from six aquatic macrophyte species collected in the Pantanal biome. Distilled water and Tween 20 solution 0.25% (*v*/*v*) were used as controls for aqueous and methanolic extracts, respectively. Values are mean ± SE. Uppercase letters compare aqueous and methanolic extracts within each treatment; lowercase letters compare treatments within each extract. Means followed by the same letter do not differ by Scott-Knott test (*p* < 0.05).

**Figure 3:**
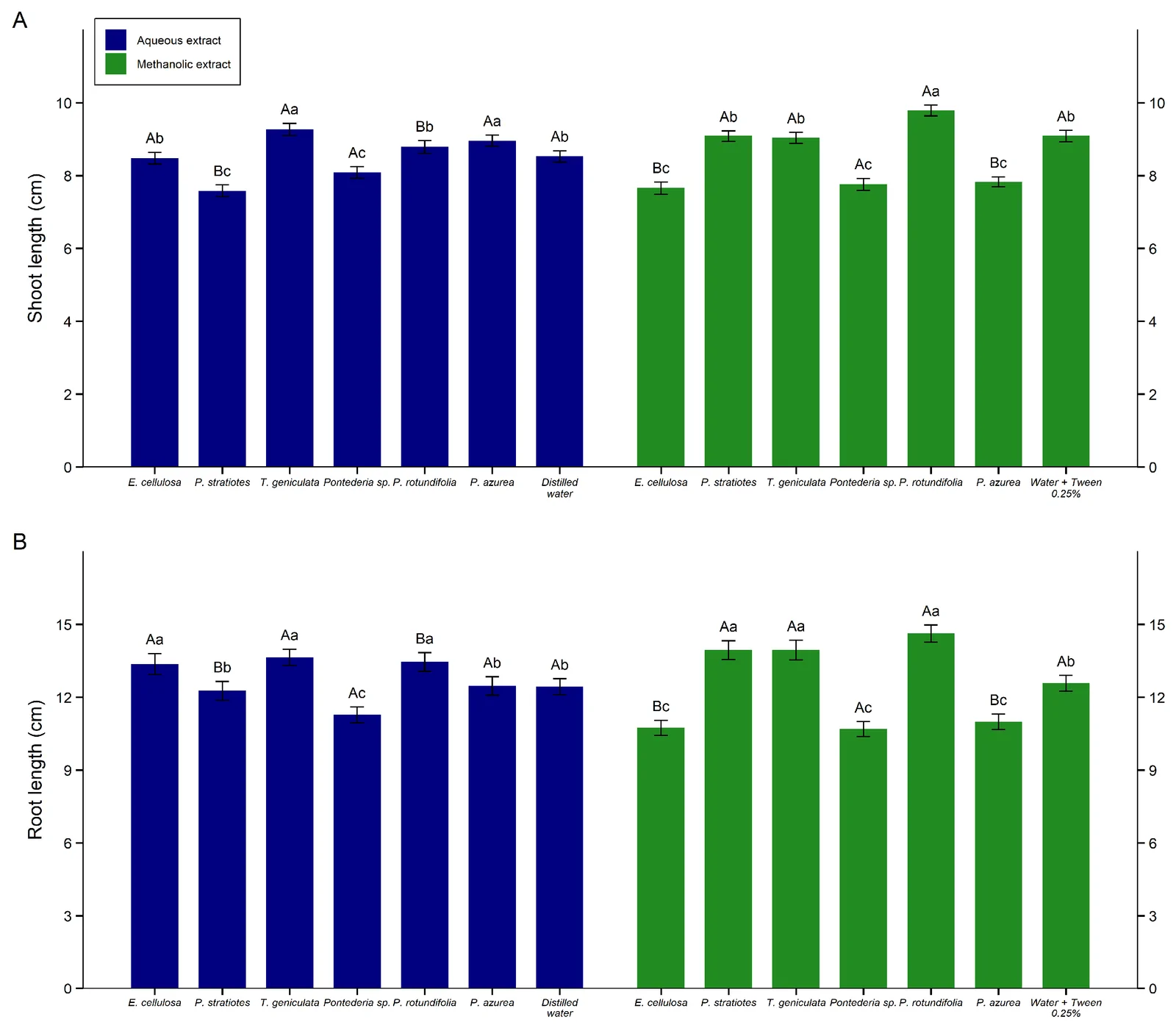
Shoot length (**A**) and root length (**B**) of soybean seedlings after treatment of seeds with aqueous and methanolic crude extracts obtained from six aquatic macrophyte species collected in the Pantanal biome. Distilled water and Tween 20 solution 0.25% (*v*/*v*) were used as controls for aqueous and methanolic extracts, respectively. Values are mean ± SE. Uppercase letters compare aqueous and methanolic extracts within each treatment; lowercase letters compare treatments within each extract. Means followed by the same letter do not differ by Scott-Knott test (*p* < 0.05).

**Figure 4:**
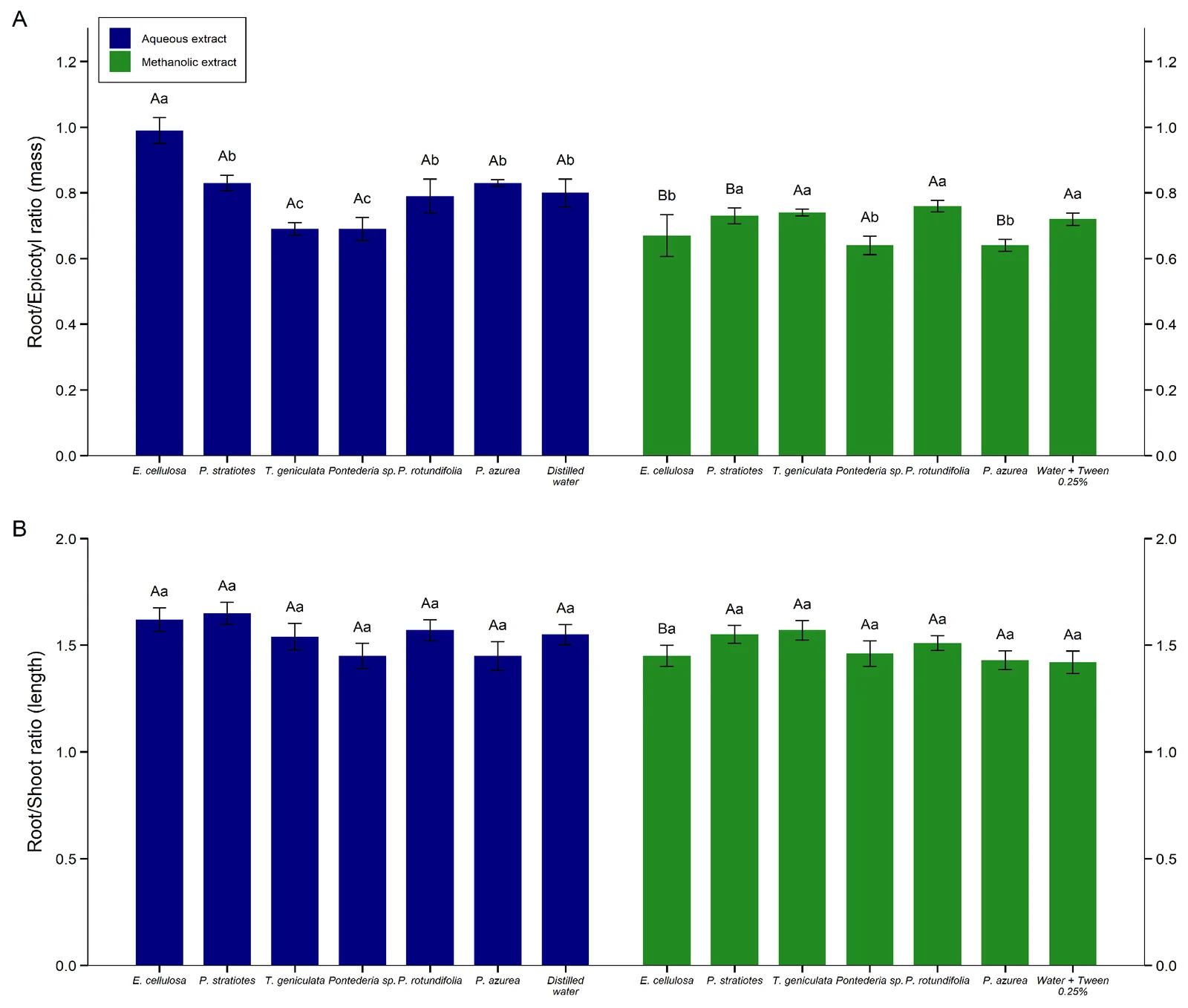
Root/Epicotyl ratio based on dry mass (**A**) and Root/Shoot ratio based on length (**B**) of soybean seedlings after treatment of seeds with aqueous and methanolic crude extracts obtained from six aquatic macrophyte species collected in the Pantanal biome. Distilled water and Tween 20 solution 0.25% (*v*/*v*) were used as controls for aqueous and methanolic extracts, respectively. Values are mean ± SE. Uppercase letters compare aqueous and methanolic extracts within each treatment; lowercase letters compare treatments within each extract. Means followed by the same letter do not differ by Scott-Knott test (*p* < 0.05).

**Figure 5:**
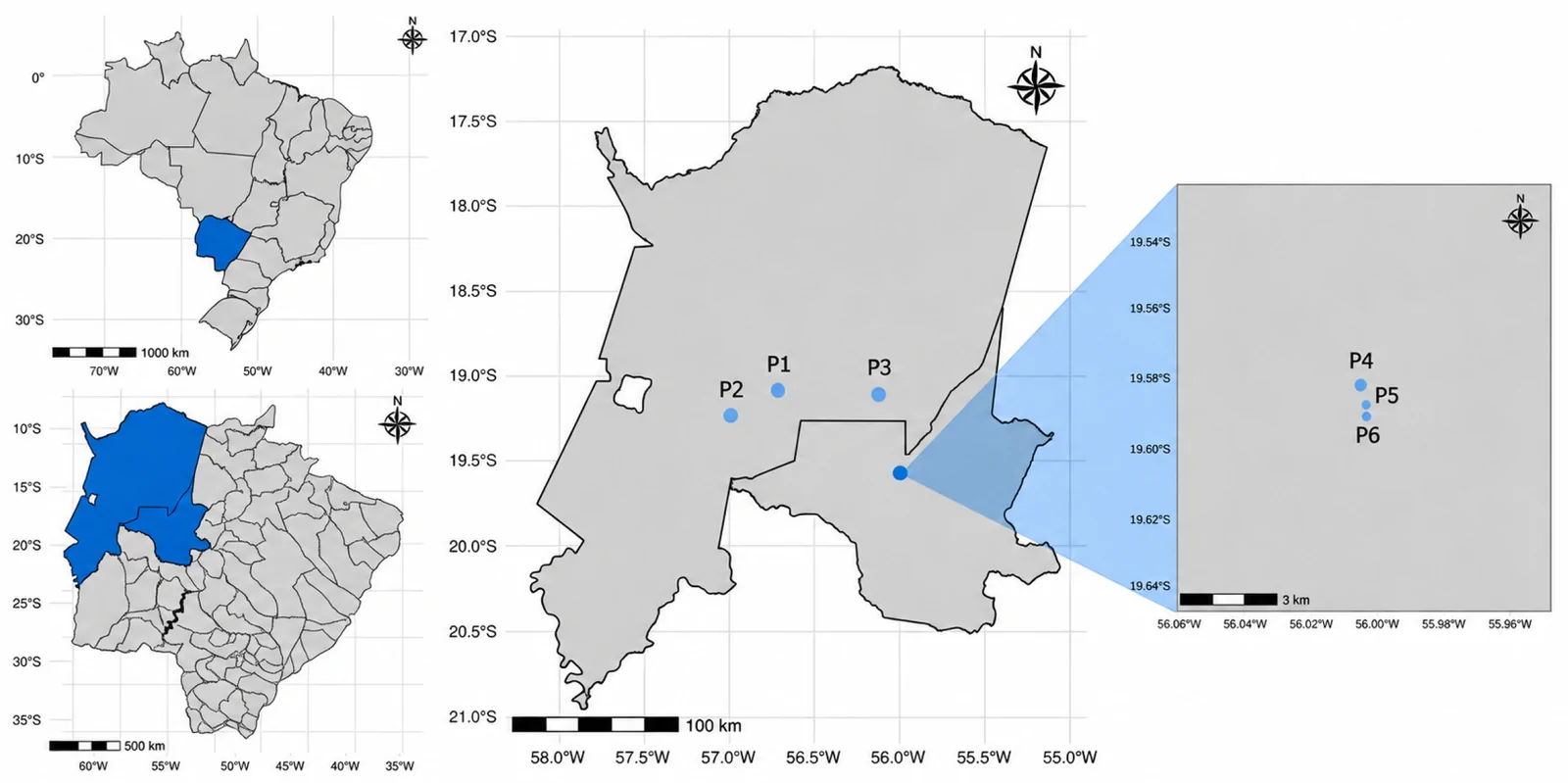
Geographic location of the study area and collection sites in the Pantanal biome, Mato Grosso do Sul, Brazil. The dark blue areas in the smaller maps indicate the location of the study area within Brazil and Mato Grosso do Sul state. The gray areas in the main maps represent the study region. The light blue dots (P1–P6) indicate the sampling points.

**Table 1:** Dry biomass yield of Pantanal aquatic macrophytes after drying (forced-air oven, 45 °C, until constant weight) and milling (knife mill, 30-mesh sieve).

Species	Dry Biomass (kg)	Milled Biomass (kg)	Yield (%)	Residual Moisture (%)
*Eleocharis cellulosa*	3.26	2.90	88.96	6.04
*Pistia stratiotes*	2.06	1.96	95.15	8.41
*Thalia geniculata*	6.66	6.00	90.09	5.04
*Pontederia* sp.	1.22	1.10	90.16	7.49
*Pontederia rotundifolia*	1.46	1.38	94.52	5.89
*Pontederia azurea*	2.50	2.30	92.00	4.18

**Table 2:** Extraction yield (%, *w*/*w*) of aqueous and methanolic extracts and filtration residue retention from six Pantanal aquatic macrophytes. Values are mean ± sample standard deviation (SD) on a constant dry weight basis.

	Aqueous Extracts		Methanolic Extracts	
	Yield	Retention	Yield	Retention
Species	(%)			
*Eleocharis cellulosa*	8.82 ± 0.18	86.54 ± 2.05	4.84 ± 0.57	90.05 ± 0.19
*Pistia stratiotes*	11.70 ± 0.25	80.39 ± 2.66	5.11 ± 0.11	93.37 ± 0.60
*Thalia geniculata*	10.84 ± 0.27	84.97 ± 2.59	10.11 ± 0.61	86.53 ± 0.20
*Pontederia* sp.	14.96 ± 0.27	78.15 ± 0.14	4.16 ± 0.38	87.92 ± 0.61
*Pontederia azurea*	12.19 ± 0.88	83.36 ± 0.66	10.91 ± 0.39	85.12 ± 0.39
*Pontederia rotundifolia*	21.37 ± 0.93	67.50 ± 0.28	10.07 ± 0.38	84.31 ± 0.80

**Table 3:** Main infrared absorption bands (FTIR) and general chemical profiles of the aqueous and methanolic extracts obtained from the six-plant species. (*) The band at 1740 cm^−1^ is attributed to the stretching of ester carbonyls and was observed exclusively in the methanolic extracts of *Pistia stratiotes* and *Thalia geniculata*.

	Aqueous Extract		Methanolic Extract	
Species	Main Bands (cm^−1^)	Chemical Profile	Main Bands (cm^−1^)	Chemical Profile
*Eleocharis cellulosa*	3281, 1621, 1393, 1007	Polysaccharides, carbonyls	3339, 2923, 1580, 1514, 1050	Flavonoids and phenols
*Pistia stratiotes*	3278, 2919, 1638, 1027	Polysaccharides, organic acids	3382, 2921/2850, 1740 *, 1651, 1076	Esters and lipids
*Pontederia sp*	3280, 2919, 1590, 1023	Polysaccharides, aromatics	3300, 2918, 1589, 1397, 1054	Flavonoids and phenols
*Thalia geniculata*	3254, 2918/2849, 1608, 1054	Polysaccharides, aliphatics	3308, 2921/2851, 1740 *, 1605, 1516, 1034	Esters, lipids and flavonoids
*Pontederia azurea*	3257, 1597, 1404, 1072	Polysaccharides, aromatics/phenols	3234, 2922/2851, 1602, 1539, 1028	Flavonoids and phenols
*Pontederia rotundifolia*	3273, 2921, 1587, 1397, 1063	Polysaccharides, aromatics/phenols	3276, 2923, 1583, 1397, 1053	Flavonoids and phenols

**Table 4:** Summary of two-way ANOVA for germination and seedling growth variables of soybean seeds treated with aqueous and methanolic extracts from six Pantanal aquatic macrophytes. Treatment = species effect; Extraction = aqueous vs. methanolic effect; T × E = interaction between treatment and extraction. CV = coefficient of variation. Degrees of freedom: Treatment = 5; Extraction = 1; T × E = 5; Residuals = 33; *n* = 4.

Source of Variation	G (%)	GSI	EDM (g)	CDM (g)	RDM (g)	SL (cm)	RL (cm)	R/ER	R/SR
Treatment	**	**	***	**	***	***	***	***	*
Extraction	ns	ns	ns	**	***	ns	ns	***	ns
T × E	ns	ns	**	***	***	***	***	***	ns
CV (%)	4.92	6.42	7.99	6.26	9.49	17.72	27.58	8.13	33.43

Note: G = Germination percentage; GSI = Germination Speed Index; EDM = Epicotyl dry mass; CDM = Cotyledon dry mass; RDM = Root dry mass; SL = Shoot length; RL = Root length; R/ER = Root/Epicotyl ratio (mass); R/SR = Root/Shoot ratio (length). Significance codes: *** *p* < 0.001; ** *p* < 0.01; * *p* < 0.05; ns = not significant (*p* ≥ 0.05).

**Table 5:** Geographic coordinates, collection dates, and aquatic species sampled in the Nhecolândia sub-region of the Pantanal, Mato Grosso do Sul, Brazil, in November 2025.

Points	(°S)	(°W)	Date	Time	Species
P1	19.07185	56.71957	04 Nov	07:18:54	*Thalia geniculata*
P2	19.21742	56.99471	04 Nov	10:37:54	*Pontederia* sp.; *Eleocharis cellulosa*
P3	19.11291	56.12679	05 Nov	07:57:51	*Eleocharis cellulosa*
P4	19.58021	56.00619	05 Nov	10:10:45	*Pontederia azurea*
P5	19.58701	56.00341	05 Nov	10:55:28	*Pontederia rotundifolia*; *E. cellulosa*
P6	19.59045	56.00357	06 Nov	08:56:39	*Pistia stratiotes*

**Table 6:** Concentrations on a constant dry weight basis (mg mL^−1^), of aqueous and methanolic extracts obtained from Pantanal aquatic macrophytes applied to soybean seeds.

Species	Aqueous Extract	Methanolic Extract
	mg mL^−1^	
*Thalia geniculata*	11.28	10.35
*Pontederia* sp.	4.15	8.84
*Eleocharis cellulosa*	9.23	5.38
*Pontederia azurea*	10.15	12.45
*Pontederia rotundifolia*	12.10	10.16
*Pistia stratiotes*	13.11	5.23

## Data Availability

The original contributions presented in this study are included in the article/Supplementary Materials. Further inquiries can be directed to the corresponding author.

## Supplementary Materials

The following supporting information can be downloaded at https://www.mdpi.com/article/10.3390/plants15213429/s1: Figure S1: Visual appearance of milled biomass from six aquatic macrophyte species collected in the Pantanal biome, after drying and knife milling through a 30-mesh sieve; Figure S2: Aqueous (A) and methanolic (B) crude extracts from six Pantanal macrophytes after drying; Figure S3: Original FTIR spectra of aqueous (A) and methanolic (B) extracts from *Eleocharis cellulosa*; Figure S4: Original FTIR spectra of aqueous (A) and methanolic (B) extracts from *Pistia stratiotes*; Figure S5: Original FTIR spectra of aqueous (A) and methanolic (B) extracts from *Thalia geniculata*; Figure S6: Original FTIR spectra of aqueous (A) and methanolic (B) extracts from *Pontederia* sp.; Figure S7: Original FTIR spectra of aqueous (A) and methanolic (B) extracts from *Pontederia rotundifolia*; Figure S8: Original FTIR spectra of aqueous (A) and methanolic (B) extracts from *Pontederia azurea*.
