# Supplementary material for: Bioprospecting Pantanal Aquatic Macrophytes as Sources of Plant-Derived Biostimulants for Soybean Seed Treatment
Source: Plants (Basel). 2026 Nov 4;15(21):3429. doi: 10.3390/plants15213429 (PMC13765932; doi:10.3390/plants15213429)
Supplement: Supplementary file 1 [file plants-15-03429-s001.zip › plants-4547149-supplementary.pdf]

plants

MDPI

Supplementary Materials

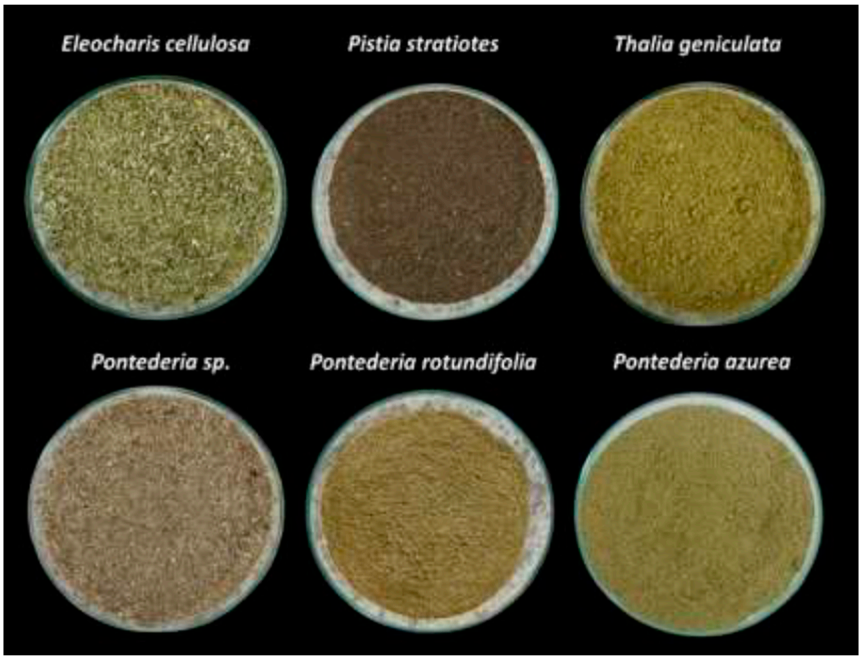

Figure S1. Visual appearance of milled biomass from six aquatic macrophyte species collected in the Pantanal biome, after drying and knife milling through a 30-mesh sieve.

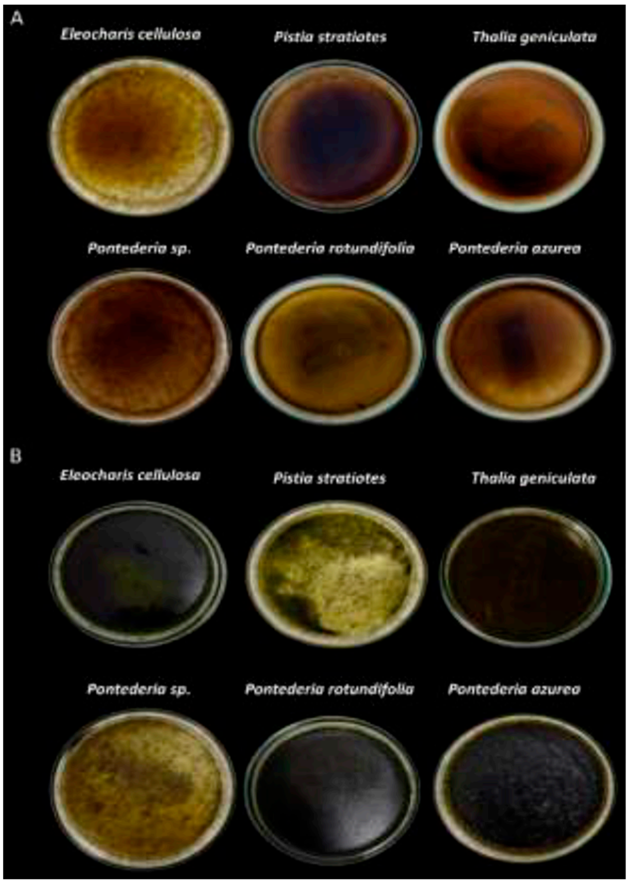

Figure S2. Aqueous (A) and methanolic (B) crude extracts from six Pantanal macrophytes after drying.

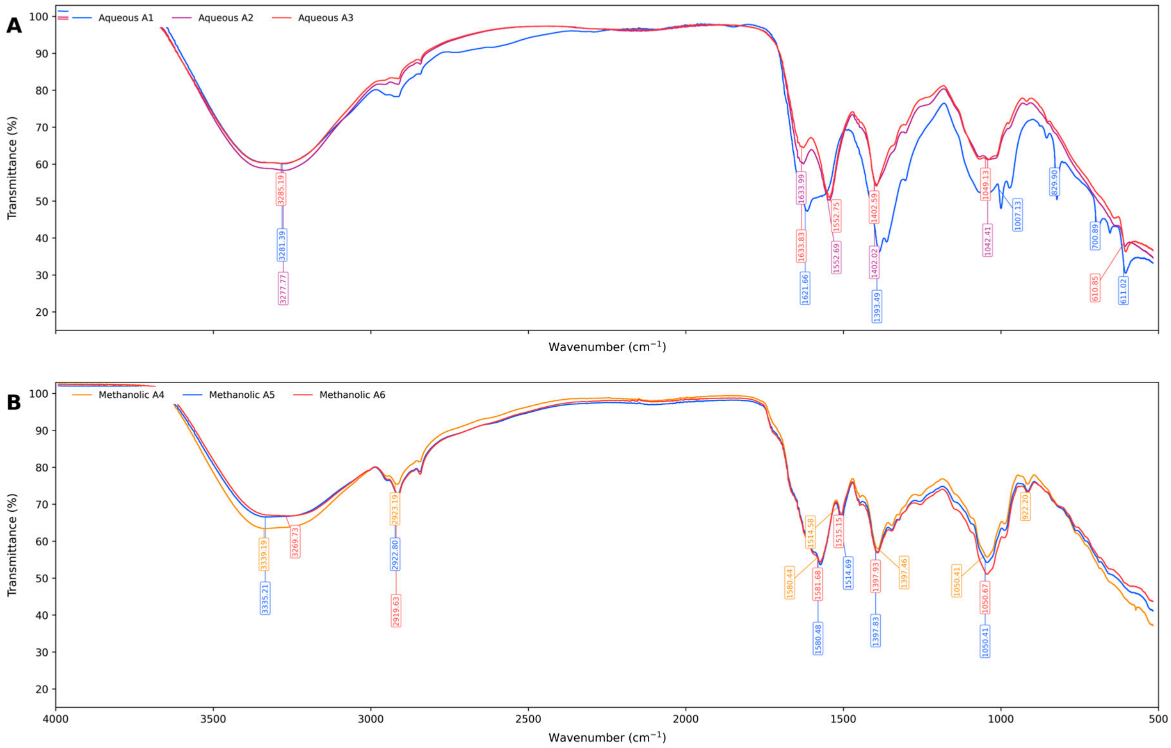

Figure S3. Original FTIR spectra of aqueous (A) and methanolic (B) extracts from *Eleocharis cellulosa*.

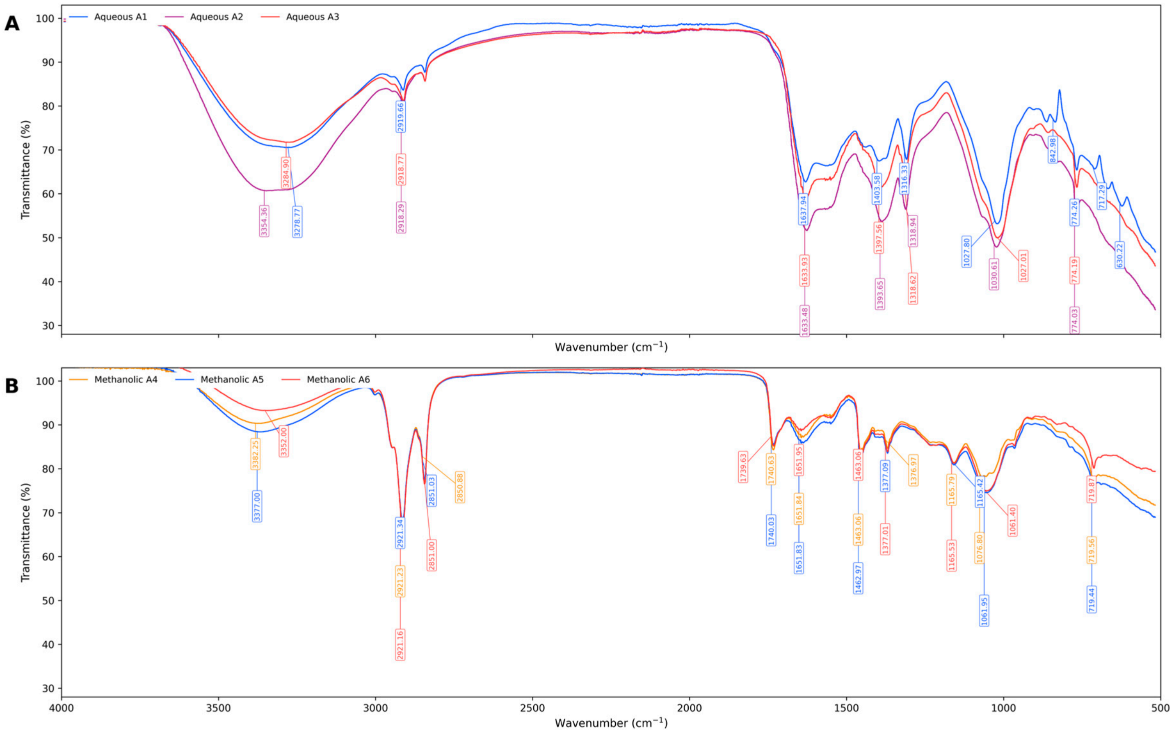

Figure S4. Original FTIR spectra of aqueous (A) and methanolic (B) extracts from *Pistia stratiotes*.

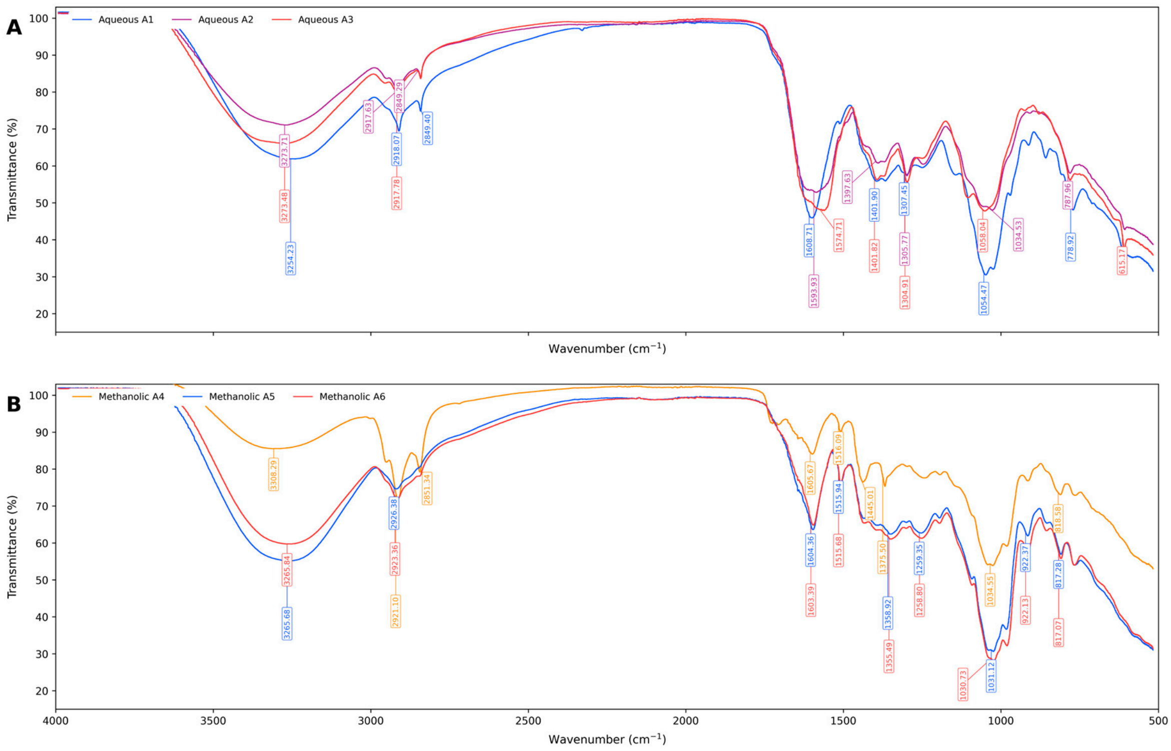

Figure S5. Original FTIR spectra of aqueous (A) and methanolic (B) extracts from *Thalia geniculata*.

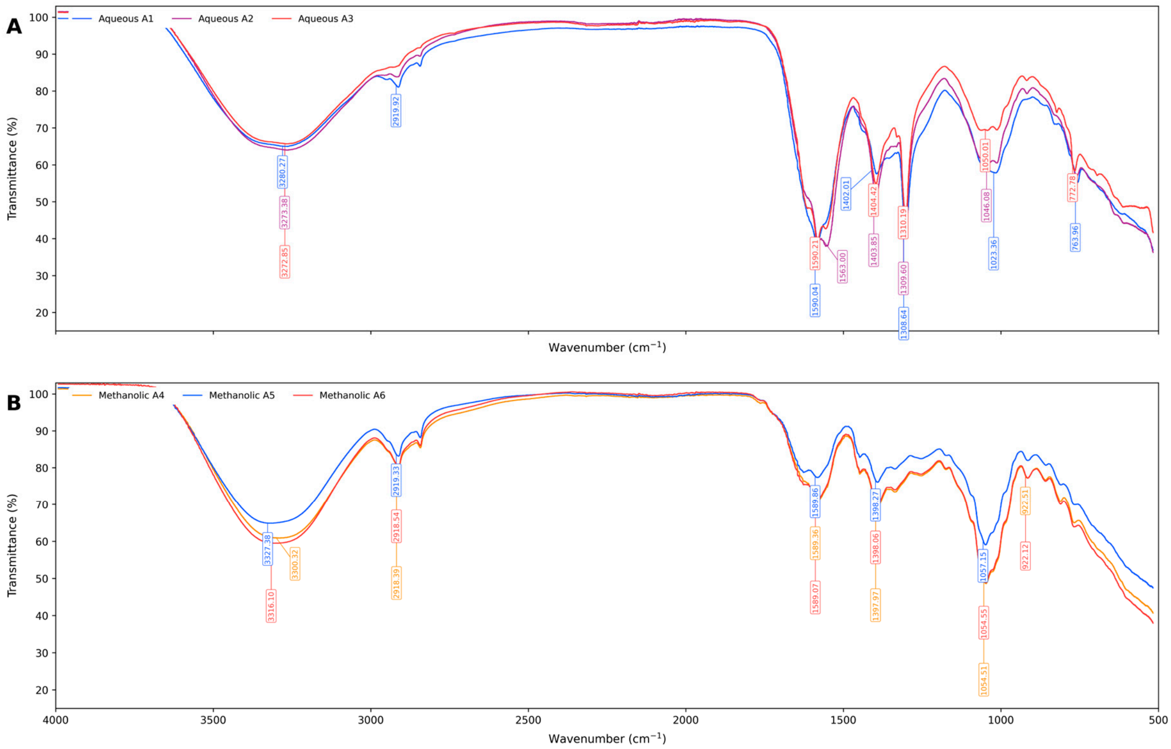

Figure S6. Original FTIR spectra of aqueous (A) and methanolic (B) extracts from *Pontederia* sp.

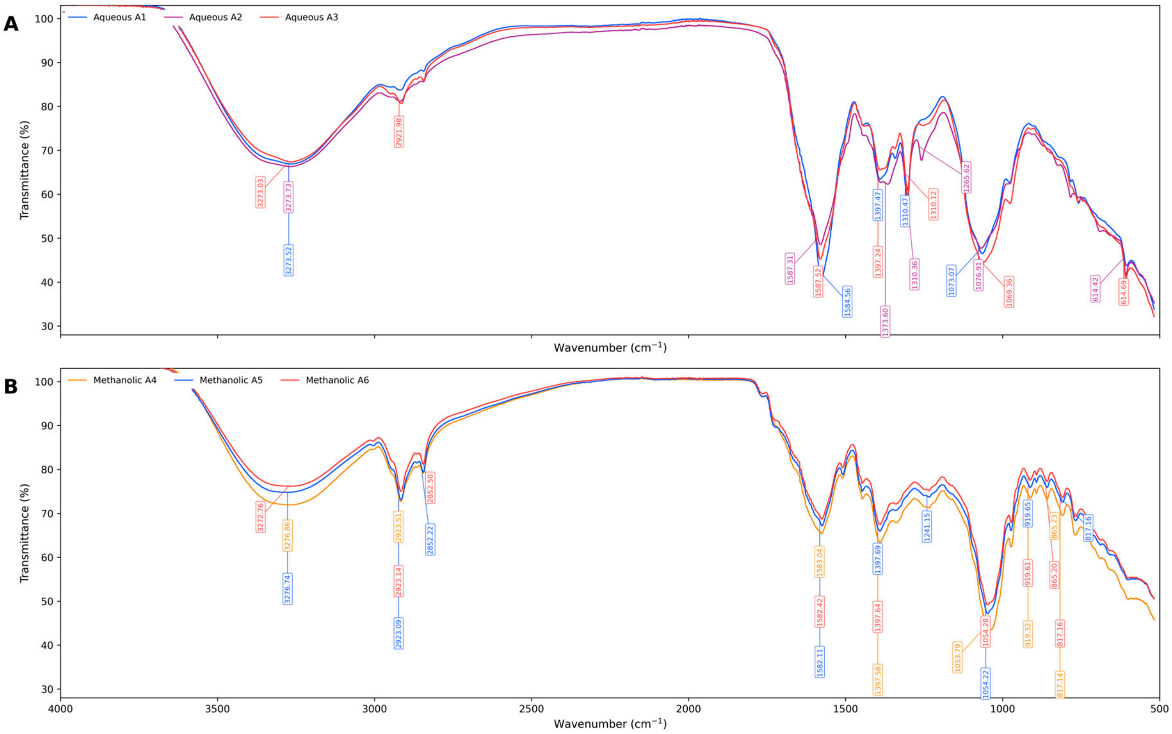

Figure S7. Original FTIR spectra of aqueous (A) and methanolic (B) extracts from *Pontederia rotundifolia*.

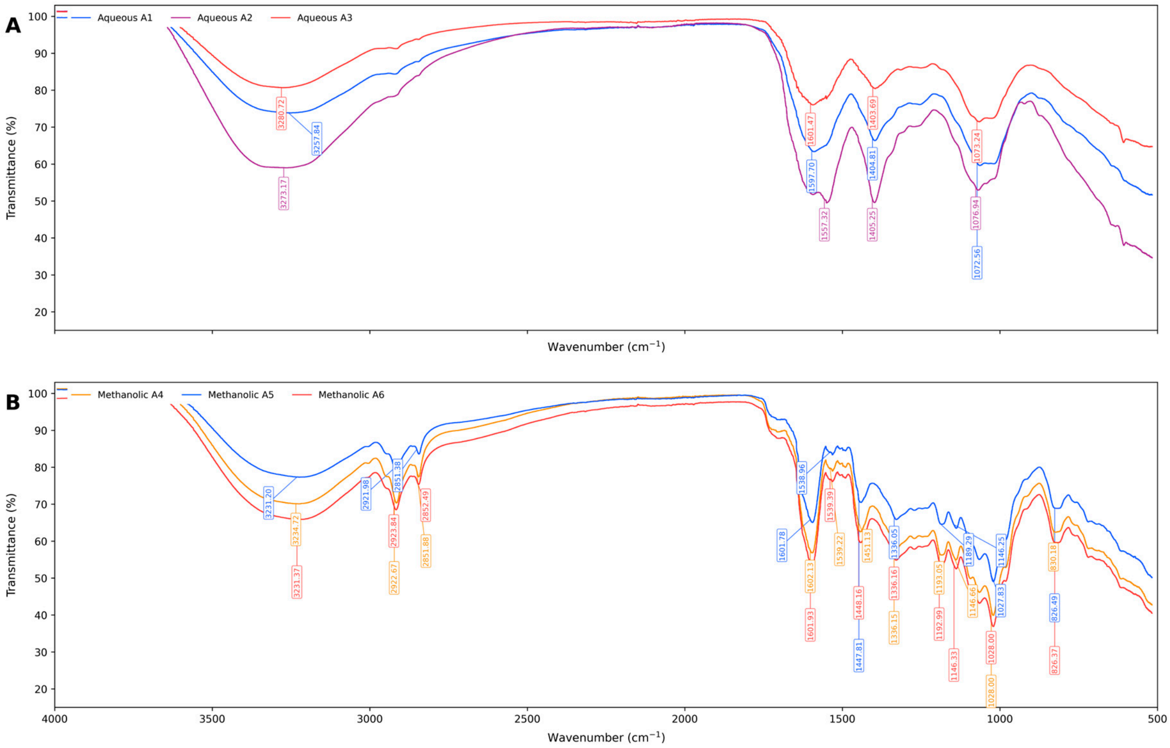

Figure S8. Original FTIR spectra of aqueous (A) and methanolic (B) extracts from *Pontederia azurea*.
